# Kineplastic Amputation

**Published:** 1918-10

**Authors:** 


### ORTHOPEDICS

***Kineplastic Amputation.*** By V. Putti, Director of the Rizzoli Institute at Bologna. Abstract of an address before the Royal Society of Medicine. *The Lancet,* June 8, 1918.

Professor Putti expressed his great confidence in the beneficial results to be obtained by the methods of kinematization based on the theories of plastic-motor (motor flaps originally advocated by Professor Giuliano Vanghetti during the second Italian expedition into Abyssinia in 1896). Germany and Austria have given the theory and its practical application a very careful consideration, but hardly a mention of it is made in the publications of France, England and America. Before the present war even in Italy kineplastic amputations did not exceed twenty.

The terms *kinematic plastics* or *kineplastics* are given by Dr. Vanghetti to any bloodless or operative plastics that tend to economize, restore, or substitute muscular masses which can be employed towards imparting direct and voluntary movement to an artificial limb.

Every moving entity obtained kineplastically is called *plastic-motor* (*motor-flaps*). This term denotes the living part of the apparatus which moves the artificial limb.

The principle on which the creation of such motor flaps is based is that the tendon and muscle, provided they have the necessary physiological protection (skin, vessels, nerves, etc.) can generally be used for the kinematic prothesis, provided also that they admit of the formation of an artificial point of attachment. Kinematization can be effected or prepared for at the time of the amputation. It can also be accomplished on stumps that have already healed.

Motor flaps may vary as to their number, position, shape, and function. The most elementary and the most commonly used are the *clava* and *ansa* motors, and those obtained by canalizing, or tunnellizing, of the muscular masses. In a single prothesis there may be one or more such motors, acting together or separately, and even opposite movements. The upper limb is the one most frequently kinematized.

*Technic.* For the purpose of kinematization more bone and soft tissue must be spared at the time of operation than has hitherto been thought necessary. Even if the pressure of work is too great to permit the completion of kinematic surgery, at least the operator may amputate in a manner to make possible later kineplastic treatment. This should be done so as not to interfere with the ordinary dressing of the wound. When there is no more inflammation or fear of complications, the motor flaps may be prepared.

The *motor flap* should be planned to withstand a firm, resisting, and painless grip, and also a traction that may attain a high degree. It must also be provided with a sufficient amount of functional muscular tissue. To this end the motors must be covered with skin in perfect condition, well nourished and provided with a normal degree of sensibility. In shape and dimensions they must be large enough to permit the attaching of the necessary hooks, wings, and rods. Muscular masses should be chosen which will produce broad, strong, and independent contractions. The tendon is best suited to the transmission of muscular traction and should, when possible, be employed in forming motor flaps.

If tendons are not available, muscular bundles may be included in the motor flaps, or the muscular masses may be tunnellized in order to obtain extra-terminal motors; that is, a means of purchase outside of the stump itself. The antagonistic powers necessary to all active movement must be provided either from the stump itself, or by means of elastic resistance in the artificial limb. To provide the motor with all requisites the various forms of grafting known to plastic surgery may be employed.

By kinematization of the quadriceps, the stump may be made to control the extension of the knee and to restrain flexion, thus overcoming one of the most difficult problems of prothesis.

By kinematization, even the stumps which formerly were considered useless may be made serviceable, as, for instance, the carpal stumps, short forearm stumps, and disarticulation stumps. If, in shoulder disarticulation, the deltoid and pectoralis major may be spared and covered with skin, and a point of attachment provided, a considerable functional result is possible. Points of attachment provided in short forearm and leg stumps may be created to correspond to the insertion of the biceps and patella tendons.

When a point of attachment is not easily available at the end of the stump, one may be reached by canalization through the muscle mass. The canal, if properly coated with skin, may tolerate the metallic rod which conveys the muscular activity to the artificial limb.

A flap to be of practical value must have a power of 10 kilogramme-centimeters and must shorten not less than one inch. Power of as much as 100 kilogramme-centimeters has been developed. The flap easily supports the necessary constriction of ring and screw, which attaches the communicating mechanism without pain or impairment of blood supply. The author in the case of one thigh stump obtained a contraction of 2-1/5 inches and strength to lift 44 pounds.

The speaker exhibited various casts showing the types of motor flaps, which he had himself made, and kinematograph representations of the functional value of this type of prothesis. No supplementary shoulder or hip movement is required as in the older forms of prothesis. The physician and mechanic must act in close co-operation to produce a limb perfectly adapted to the nature of the stump. When this is done, the results obtained in practice amply justify all the claims made in behalf of this method.

